# Endothelial-to-Mesenchymal Transition Mechanisms in Vascular Remodeling of Pulmonary Hypertension

**DOI:** 10.3390/ijms27114951

**Published:** 2026-05-29

**Authors:** Xinyi Chen, Juan Su, Huihui Liu, Yajing Qin, Mengyao Li, Peili Xie

**Affiliations:** 1 College of Clinical Medicine, Affiliated Hospital of Qinghai University, Xining 810007, China; 13993626516@163.com (X.C.);; 2 Department of Rheumatology, Affiliated Hospital of Qinghai University, Xining 810007, China

**Keywords:** pulmonary arterial hypertension, PAH, endothelial-to-mesenchymal transition, EndMT, vascular remodeling, hypoxia, therapeutic strategies

## Abstract

Pulmonary arterial hypertension (PAH) is a chronic and progressive cardiopulmonary vascular disorder associated with poor clinical prognosis. Its hallmark pathological feature is sustained elevation of pulmonary vascular resistance resulting from extensive vascular remodeling. Endothelial-to-mesenchymal transition (EndMT), a critical event driving vascular remodeling, is increasingly recognized as central to PAH development and progression. This review systematically outlines the convergence of multiple pathophysiological insults on endothelial dysfunction and intimal remodeling in PAH, highlighting their roles in initiating EndMT. Principal factors include: (1) genetic and molecular alterations, such as BMPR2 mutations and epigenetic dysregulation; (2) environmental and toxic exposures, including chronic hypoxia and anorexigens; (3) inflammatory and immune dysregulation, exemplified by chronic inflammatory infiltrates and autoimmune conditions; and (4) hemodynamic and metabolic disturbances, notably aberrant shear stress and lipid metabolic imbalance. Given the critical contribution of EndMT to PAH pathogenesis, therapeutic strategies aimed at reversing EndMT represent promising anti-remodeling interventions. Preclinical studies have begun exploring EndMT-targeted therapies, including mesenchymal stem cell (MSC) transplantation and dipeptidyl peptidase-4 (DPP-4) inhibitors. Herein, we summarize recent advances regarding EndMT in PAH, dissect the molecular drivers and modulators initiating and sustaining EndMT, and critically evaluate emerging therapeutic strategies harnessing this pathway for clinical benefit.

## 1. Introduction

Pulmonary arterial hypertension (PAH) is a chronic and progressive cardiopulmonary vascular disorder characterized pathologically by sustained pulmonary vascular remodeling and persistently elevated pulmonary vascular resistance, and clinically by right ventricular (RV) hypertrophy that may ultimately progress to right heart failure, the leading cause of mortality in PAH. PAH has become a major global public health concern; registry data from high-income countries report an incidence of approximately 6 cases per million and a prevalence of 48–55 cases per million adults [1]. Current targeted therapies primarily induce pulmonary vasodilation; however, because they do not reverse established vascular remodeling, they fail to address the underlying pathology, and the prognosis of PAH remains poor.

Pulmonary vascular remodeling is the central pathological event in PAH. It elevates pulmonary vascular resistance and subsequently increases mean pulmonary arterial pressure [2]. Histologically, this remodeling is characterized by intimal hyperplasia, medial hypertrophy, and adventitial fibrosis. Numerous studies have identified endothelial-to-mesenchymal transition (EndMT) as a critical driver of these structural alterations [3].

EndMT is a dynamic process in which endothelial cells, under physiological or pathological conditions and in response to stimuli such as transforming growth factor-β (TGF-β), oxidative stress, and inflammation, lose their endothelial phenotype and acquire mesenchymal characteristics. This transition involves disruption of intercellular junctions, degradation of the basement membrane, loss of cell polarity and adhesion, and a morphological shift from a cobblestone-like to a spindle-shaped appearance. These changes are accompanied by down-regulation of endothelial markers (e.g., CD31, VE-cadherin, von Willebrand factor) and up-regulation of mesenchymal markers (e.g., α-smooth muscle actin, fibroblast-specific protein 1, N-cadherin), along with enhanced proliferative, migratory, and collagen-synthetic capacities [4]. In cardiovascular biology, EndMT has been implicated in a broad spectrum of diseases, including atherosclerosis [5,6], valvular heart disease [7,8,9], cardiac fibrosis, and myocardial infarction [10]. Recent studies have further linked EndMT to both the initiation and progression of PAH [11,12]. The present review synthesizes recent advances by focusing on pathogenic cues, such as hypoxic signaling, inflammatory cascades, BMPR2 dysregulation, and redox imbalance, that couple EndMT to PAH development. We integrate these findings to delineate the molecular targets and interacting factors through which EndMT contributes to PAH pathogenesis and to highlight therapeutic strategies aimed at modulating EndMT for clinical benefit. This review synthesizes recent mechanistic insights that connect hypoxia, immune signaling, metabolic reprogramming, and oxidative stress with EndMT in PAH, while emphasizing emerging therapeutic strategies directed against these pathways.

## 2. Pathophysiology of PAH

**Figure 1:**
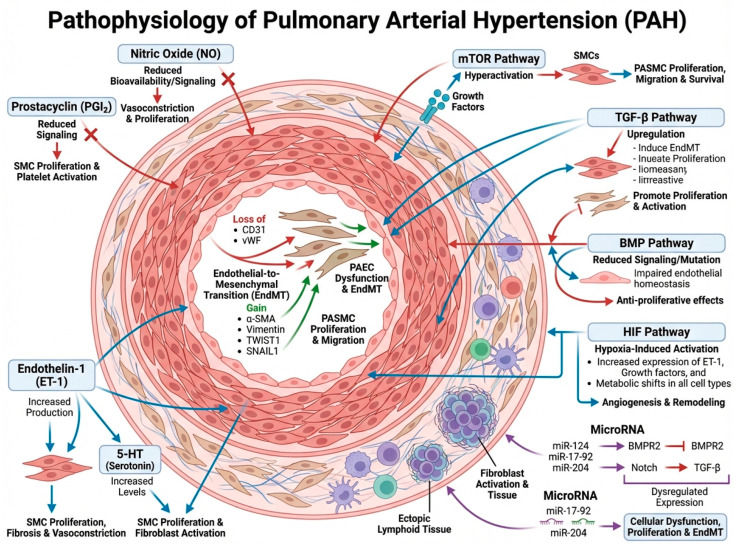
This figure systematically illustrates the multi-pathway molecular mechanisms underlying the development and progression of pulmonary arterial hypertension (PAH). Centered on the pulmonary arteriole, the diagram highlights multiple cell types—such as pulmonary arterial endothelial cells, smooth muscle cells, and fibroblasts—and their pathological alterations in PAH. Key signaling pathways involved include: **NO/PGI_2_ pathway**: Diminished nitric oxide (NO) and prostacyclin (PGI_2_) signaling leads to vasoconstriction, smooth muscle cell (SMC) proliferation, and increased platelet activation. **Endothelin-1 (ET-1) and 5-hydroxytryptamine (5-HT) pathway**: Elevated levels of ET-1 and 5-HT promote SMC proliferation, fibrosis, and vasoconstriction. **TGF-β pathway**: Upregulated TGF-β signaling induces endothelial-mesenchymal transition (EndMT), cell proliferation, and activation, thereby contributing to vascular remodeling. **BMP pathway**: Reduced or mutated bone morphogenetic protein (BMP) signaling impairs endothelial homeostasis and diminishes anti-proliferative effects. **mTOR pathway**: Hyperactivation of mTOR signaling enhances SMC proliferation, migration, and survival. **HIF pathway**: Activation of hypoxia-inducible factor (HIF) upregulates the expression of ET-1, TGF-β, and other factors, promoting angiogenesis and vascular remodeling. **MicroRNA regulation**: Various miRNAs (e.g., miR-124, miR-17-92, miR-204) participate in cellular dysfunction, proliferation, and EndMT by modulating signaling pathways such as BMPR2, Notch, and TGF-β. Furthermore, the figure depicts critical pathological events—including endothelial dysfunction, EndMT, PASMC proliferation and migration, fibroblast activation, and ectopic lymphoid tissue formation—which collectively drive vascular remodeling and disease progression in PAH. Arrows denote the direction of signaling or cellular effects; red arrows indicate pathogenic/promoting effects, blue arrows indicate signaling crosstalk or downstream transmission, green arrows indicate phenotypic transition, and purple arrows indicate microRNA-mediated regulation. Red crosses represent reduced signaling or loss of protective activity, and blunt-ended lines indicate inhibitory regulation. Color coding is used to improve visual distinction of signaling categories and does not necessarily indicate pathway hierarchy or strength of effect.

The pulmonary circulation is characterized by high flow and low pressure, receiving the entire cardiac output from the right ventricle. Pulmonary arteries transport deoxygenated blood from the right ventricle to the pulmonary capillaries for gas exchange. In PAH, chronic hypoxia, in situ thrombosis, and inflammation drive pulmonary vascular remodeling, ultimately leading to RV failure as the disease progresses [13]. Histopathological alterations in PAH involve all three layers of the pulmonary arterial wall: the intima, media, and adventitia. The intima primarily contains vascular endothelial cells, which can undergo EndMT under pathological conditions in PAH [14]. The pulmonary arterial media is predominantly composed of smooth muscle cells, typically exhibiting hyperproliferative phenotypes in PAH. The adventitia, a heterogeneous layer consisting of fibroblasts and inflammatory cells, commonly displays altered fibroblast phenotypes, perivascular inflammation, and ectopic lymphoid aggregates in PAH [15]. Due to these diverse pathogenic inputs, PAH pathophysiology is highly complex, involving intricate crosstalk among nitric oxide, prostacyclin, endothelin-1 (ET-1), and 5-hydroxytryptamine signaling pathways, epigenetic susceptibility, mitochondrial metabolic dysfunction, and inflammation [16]. However, irrespective of the initiating stimulus, the central pathological hallmark remains pulmonary artery endothelial cell (PAEC) dysfunction accompanied by proliferation and migration of pulmonary artery smooth muscle cells (PASMCs) [17] (Figure 1).

### 2.1. Pulmonary Vascular Remodeling in PAH

Vascular remodeling represents the fundamental pathological process in PAH, characterized by structural modifications of the intima, media, and adventitia. These changes involve proliferation and phenotypic transition of PAECs and PASMCs, coupled with intricate interactions between pulmonary artery fibroblasts (PAFs) and the extracellular matrix (ECM) within the adventitial layer [18].

#### 2.1.1. Intimal Remodeling in Pulmonary Vascular Restructuring

The intima, the innermost layer of the pulmonary arterial wall, comprises endothelial cells and a thin subendothelial compartment, making it the thinnest among the three vessel layers (intima, media, and adventitia). Endothelial cells, together with the underlying basal lamina, form a selectively permeable barrier controlling fluid, gas, and macromolecule exchange. The luminal endothelial surface is smooth, minimizing resistance to blood flow, and these cells release substances that inhibit platelet aggregation (prostacyclin [PGI_2_], nitric oxide [NO]) and prevent thrombus formation through fibrinolytic (t-PA), anticoagulant (heparan sulfate), and antithrombotic (thrombomodulin/protein C system) mechanisms. Additionally, endothelial-derived mediators regulate vascular smooth muscle tone, suppress atherogenesis, and limit vascular-wall thickening. Following injury or pathological stimuli, endothelial cells secrete inflammatory mediators involved in vascular repair and contribute to pathological processes such as atherosclerosis.

Under physiological conditions, PAECs provide a broad, unobstructed luminal surface that helps maintain the low perfusion pressure characteristic of the pulmonary circulation. In severe PAH, however, intimal thickness may increase up to three-fold, which can elevate pulmonary vascular resistance by approximately 40-fold. Despite extensive investigation, the mechanisms underlying this intimal injury remain incompletely understood [19]. Normally, PAECs remain quiescent and secrete bioactive mediators that maintain vascular homeostasis. When factors that restrain PAEC and PASMC proliferation are disrupted, and when inflammatory mediators become increasingly activated, PAEC and PASMC dysfunction ensues, leading to impaired vascular function and contributing to the initiation and progression of PAH [15].

Multiple factors contribute to intimal remodeling and PAEC dysfunction in PAH, including genetic, environmental, inflammatory, and metabolic influences. These can be summarized as follows:

##### Genetic and Molecular Abnormalities

Genetic susceptibility is a major intrinsic determinant of PAEC dysfunction, arising primarily from pathogenic mutations or signaling imbalances that disrupt endothelial homeostasis.

① Gene mutations

**Figure 2:**
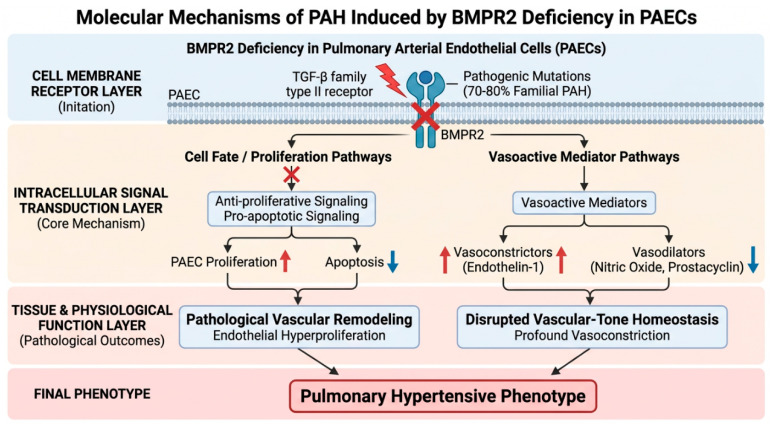
Molecular Mechanisms of PAH Induced by BMPR2 Deficiency in PAECs. The diagram illustrates how BMPR2 mutations lead to PAEC dysfunction by suppressing anti-proliferative/pro-apoptotic signals and altering the balance of vasoactive mediators. These molecular changes ultimately drive pathological vascular remodeling and vascular tone dysregulation, thereby contributing to the development of pulmonary arterial hypertension. Black arrows indicate mechanistic directionality and downstream progression; red upward arrows denote increased signaling, mediator production, or cellular responses; blue downward arrows denote decreased signaling or reduced biological effects; red crosses (×) indicate functional loss or inhibition; and the lightning symbol denotes pathogenic BMPR2 mutation.

BMPR2 mutations are the most common genetic cause of heritable PAH, accounting for 70–80% of familial cases. BMPR2 encodes a type II receptor in the TGF-β superfamily. Pathogenic variants impair anti-proliferative and pro-apoptotic signaling in PAECs while enhancing the release of vasoconstrictors such as endothelin-1 and reducing the production of vasodilators, including nitric oxide and prostacyclin. These alterations disrupt vascular-tone homeostasis [20] (Figure 2). In the study by Wen Tian, PhD, et al. [21], the establishment of a “two-hit” model of “BMPR2 mutation + inflammation” provides strong evidence for the mechanism by which BMPR2 mutations lead to hereditary pulmonary arterial hypertension (hPAH). The research confirms that BMPR2 deficiency not only induces a pro-proliferative and anti-apoptotic pathological phenotype in pulmonary arterial endothelial cells (PAECs) but also renders them highly sensitive to inflammation. This, in turn, activates the TGF-β signaling pathway, triggering endothelial-to-mesenchymal transition (EndMT) and the sustained production of inflammatory mediators (such as LTB4), thereby directly disrupting vascular homeostasis and driving pulmonary vascular remodeling. At the molecular level, this work offers a profound explanation of how BMPR2 mutations, through dysregulated signaling, convert genetic susceptibility into severe pulmonary vascular pathology.

② Other gene mutations

Variants in ACVRL1 (activin receptor-like kinase 1) and ENG (endoglin) also impair endothelial homeostasis. Both genes regulate endothelial proliferation and angiogenesis; their mutations diminish PAEC repair capacity, promote disorganized vascular architecture, and drive pathological remodeling [22].

③ Epigenetic dysregulation

**Figure 3:**
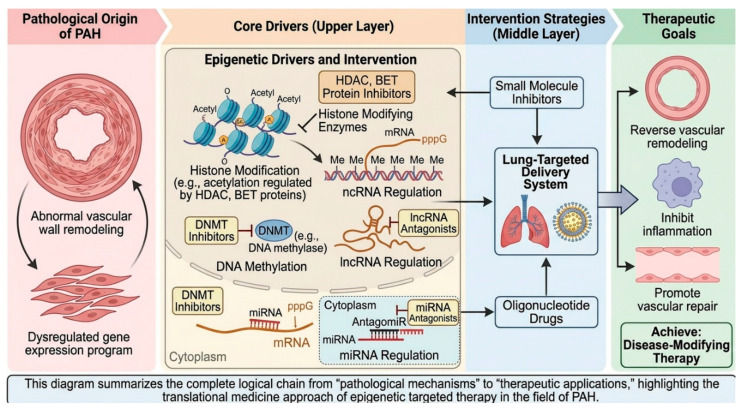
Schematic diagram of epigenetic drivers and transformative therapeutic strategies in PAH. This figure illustrates the pathological mechanisms from epigenetic abnormalities (including histone modifications, DNA methylation, and non-coding RNA regulation) to vascular remodeling, and summarizes potential therapeutic strategies targeting these drivers, including small-molecule inhibitors and lung-targeted oligonucleotide delivery systems, aiming to achieve reversal of vascular remodeling and disease-modifying therapy.

The pathological core of pulmonary arterial hypertension (PAH) lies in the abnormal remodeling of the vascular wall and the dysregulation of gene expression programs, with epigenetic processes—such as DNA methylation, histone modifications, and non-coding RNA regulation—playing a key driving role. Due to the reversible nature of these epigenetic modifications, intervening in these mechanisms using small-molecule inhibitors or oligonucleotide drugs offers novel therapeutic strategies for reversing vascular wall proliferation, hypertrophy, and inflammatory responses. Currently, preclinical studies targeting DNA methyltransferases, histone-modifying enzymes (e.g., HDACs, BET proteins), and various non-coding RNAs (miRNAs, lncRNAs) have demonstrated significant anti-remodeling and vascular repair potential in animal models, demonstrating that lung-targeted delivery of oligonucleotides to modulate gene expression programs is an effective approach for achieving disease-modifying therapy in PAH [23] (Figure 3).

##### Environmental and Toxic Exposures

Exogenous agents may directly or indirectly damage PAECs, triggering endothelial dysfunction and intimal remodeling.

① Drugs and toxins

Appetite suppressants (e.g., fenfluramine, dexfenfluramine) inhibit serotonin reuptake, causing intracellular calcium overload and oxidative stress in PAECs while suppressing endothelial nitric-oxide synthase (eNOS) activity and reducing nitric-oxide production [24].

② Drugs of abuse

Cocaine and methamphetamine directly stimulate PAECs to release endothelin-1 and impair mitochondrial function, resulting in cellular energy-metabolism disturbances [25].

③ Industrial chemicals

Organic solvents and heavy metals disrupt PAEC membrane integrity via oxidative stress and inflammatory mechanisms [25].

④ Hypoxia and high-altitude exposure

Chronic hypoxia activates hypoxia-inducible factor-1α (HIF-1α) in PAECs, increasing expression of ET-1 and VEGF while suppressing eNOS activity, thereby promoting vasoconstriction and endothelial proliferation [26]. A study by Xiaofei Shi et al. investigated the relationship between HIF-1α/VEGF signaling and disease severity in connective-tissue-disease-associated PAH (CTD-PAH) [27]. Serum HIF-1α and VEGF levels, along with hemodynamic and functional parameters, including mean pulmonary arterial pressure (mPAP), NT-proBNP, 6 min walk distance (6MWD), and pulmonary vascular resistance (PVR), were assessed. HIF-1α and VEGF expression in pulmonary tissue was significantly higher in CTD-PAH patients than in controls and correlated positively with mPAP and BNP, and negatively with 6MWD, suggesting their potential utility as diagnostic indicators in CTD-PAH.

##### Inflammation and Immune Dysregulation

Persistent inflammation and immune dysregulation cause endothelial injury and promote maladaptive vascular remodeling through multiple interconnected pathways:

① Inflammatory infiltrates

Perivascular inflammation is a hallmark histological feature observed consistently across experimental pulmonary hypertension (PH) models and in human PAH. Growing evidence indicates that these inflammatory infiltrates actively drive both initiation and progression of pulmonary vascular remodeling. Patients with PAH exhibit elevated circulating and tissue concentrations of cytokines, chemokines, and other inflammatory mediators, correlating with disease severity and clinical outcomes. Additionally, neutrophils, macrophages, dendritic cells, mast cells, and T and B lymphocytes accumulate around pulmonary vessels in PAH. These infiltrating immune cells drive phenotypic switching in endothelial, smooth-muscle, and fibroblast cell populations, increasing their sensitivity to inflammatory stimuli and amplifying local cytokine and chemokine secretion. A deeper understanding of the interactions among inflammatory cells, vascular cells, and soluble mediators is expected to guide the development of novel, safe, and effective immune-targeted therapies for PAH [28].

② Autoimmune disorders

In patients with systemic sclerosis or systemic lupus erythematosus who develop PAH, autoantibodies, particularly anti-endothelial cell antibodies, directly target PAECs, promoting apoptosis and increasing vascular permeability. These antibodies also activate the complement cascade, further exacerbating endothelial injury [29].

③ Viral infections

In HIV-associated PAH, the viral trans-activator protein Tat induces PAECs to release ET-1 while simultaneously inhibiting endothelial nitric oxide synthase (eNOS) activity. Direct viral infection of PAECs can lead to cell-cycle arrest and functional exhaustion, further compromising endothelial integrity [30].

##### Hemodynamic and Metabolic Disturbances

Aberrant shear stress and metabolic imbalances trigger endothelial dysfunction via mechanical and biochemical signaling pathways.

Elevated shear stress (e.g., from increased pulmonary blood flow due to left-heart failure or ventricular septal defects) is sensed by PAECs through integrin signaling, activating NF-κB. This activation promotes the secretion of inflammatory cytokines and ET-1, while simultaneously suppressing tissue-type plasminogen activator (t-PA) and reducing fibrinolytic capacity [31].

Conversely, low shear stress (e.g., sluggish flow distal to stenotic lesions) induces PAEC apoptosis and upregulates adhesion molecules such as VCAM-1, increasing platelet and leukocyte adhesion and consequently amplifying thrombotic risk [32].

Although the exact pathogenic mechanisms remain incompletely defined, accumulating evidence suggests that metabolic disturbances exert detrimental effects on both the pulmonary vasculature and the right ventricle (RV) [33]. Obesity, insulin resistance, impaired glucose tolerance, and metabolic syndrome are strongly associated with an elevated risk of developing PAH and accelerated progression of pulmonary vascular disease. A significant proportion of PAH patients demonstrate metabolic syndrome, insulin resistance, and elevated glycated hemoglobin (HbA1c) [34,35,36,37]. Notably, 56% of these patients exhibit previously undiagnosed glucose intolerance (HbA1c ≥ 6%), and patients with metabolic syndrome display substantially higher pulmonary vascular resistance (PVR) and mean pulmonary arterial pressure (mPAP) [38].

Metabolic profiling in PAH patients has identified dysregulated lipid metabolism, increased pro-inflammatory lipid species, elevated myocardial triglyceride content detectable by cardiac magnetic resonance imaging, and elevated concentrations of lipotoxic ceramide species [39,40].

Several studies have demonstrated that glucose-lowering agents can improve RV function and delay PAH progression. In a single-center, open-label phase II trial involving 20 patients with idiopathic or heritable PAH, eight weeks of metformin therapy significantly improved RV area change [41]. These findings indicate that metformin may enhance RV function and decrease RV lipid accumulation in PAH, highlighting the therapeutic potential of targeting lipid-metabolism dysfunction in this condition. Sodium–glucose cotransporter 2 inhibitors (SGLT-2i) have demonstrated beneficial metabolic effects across multiple organ systems, including the heart, brain, liver, and kidney [42]. Due to their pleiotropic mechanisms, SGLT-2i may offer advantages in both pulmonary hypertension due to left heart disease (PH-LHD) and PAH. Several randomized controlled trials are currently underway to evaluate the efficacy of SGLT-2i in PAH patients, representing a promising therapeutic avenue for this disorder.

##### Oxidative Stress and Endoplasmic-Reticulum (ER) Stress

An imbalance between oxidant and antioxidant systems, along with defective protein folding, can directly compromise PAEC function. Regarding oxidative stress, excessive activation of NADPH oxidase (NOX) in PAECs generates reactive oxygen species (ROS). ROS oxidatively modify and uncouple eNOS and prostacyclin synthase, thus reducing vasodilator synthesis. Concurrently, ROS disrupt endothelial junction proteins such as VE-cadherin, increasing vascular permeability and facilitating inflammatory-cell infiltration [43]. Meanwhile, ER stress arises from the accumulation of unfolded or misfolded proteins within the endoplasmic reticulum, triggering the unfolded protein response (UPR). In the pathogenesis of PAH, prolonged ER stress in PAECs leads to severe endothelial dysfunction by promoting apoptosis and exacerbating inflammatory responses. Furthermore, ER stress and oxidative stress are intricately linked; sustained ER stress can induce further ROS generation, creating a vicious cycle that accelerates endothelial injury and pulmonary vascular remodeling [44].

In early-stage PAH, PAECs exposed to damaging stimuli may undergo apoptosis. However, as the disease progresses, apoptosis-resistant PAECs with enhanced survival capacity emerge and predominate [45]. In advanced PAH, these hyper-proliferative, apoptosis-resistant PAECs contribute to the formation of plexiform lesions [46]. Emerging evidence suggests that metabolic and epigenetic reprogramming also fuel disease progression [47]. Notably, PAECs encompass distinct endothelial subpopulations, each exhibiting phenotypic variability according to their anatomic location within the pulmonary vasculature and the nature of local injurious stimuli.

#### 2.1.2. Medial Remodeling in Pulmonary Vascular Restructuring

The tunica media, located between the intima and adventitia, is the middle layer of the pulmonary arterial wall and varies structurally and functionally according to vessel type (elastic, muscular, or arteriolar). Its primary function is to regulate vascular tone and maintain hemodynamic stability. Smooth muscle cells, elastic fibers, and collagen fibers constitute its main components, with proportions differing among vessel categories. The media primarily comprises PASMCs, which regulate blood flow, provide elastic buffering, and respond to pathological stimuli. Through contraction or relaxation, PASMCs modulate luminal diameter, thereby adjusting pulmonary blood flow and resistance; for example, hypoxia-induced constriction optimizes ventilation-perfusion matching. Given their central role in mediating hypoxic pulmonary vasoconstriction, PASMCs remain a primary focus in PAH research [48]. Nevertheless, the contribution of PASMCs to pulmonary vascular remodeling is complex, and no single phenotype fully accounts for this multifaceted process.

Heterogeneity and phenotypic plasticity among PASMC subpopulations are critical to vascular function and enable adaptive responses of the pulmonary circulation to changing microenvironmental conditions [49,50]. In response to specific stimuli, PASMCs can reversibly transition from a quiescent state to either a contractile or synthetic phenotype, gaining or losing the capacity to proliferate, migrate, and produce ECM components. Growth factors and inflammatory mediators, including transforming growth factor-β (TGF-β), platelet-derived growth factor (PDGF), and angiotensin II, together with mechanical forces, epigenetic modifications, and heterogeneous ECM organization, are key regulators driving the contractile-to-synthetic transition [51,52,53]. During this phenotypic shift, PASMCs acquire migratory and proliferative properties that contribute to medial thickening and pulmonary vascular remodeling.

#### 2.1.3. Adventitial Remodeling in Pulmonary Vascular Restructuring

Traditionally underappreciated, the adventitia has recently been recognized as an early and critical site of vascular wall alteration, particularly inflammation, in PAH [54]. Growing evidence indicates that the adventitia not only contributes to the pathogenesis of pulmonary vascular disease but may also act as a primary driver of PAH. Composed mainly of a connective-tissue sheath surrounding the pulmonary artery, the adventitia contains pulmonary-artery fibroblasts (PAFs) as its principal cellular components [55]. During PAH-associated vascular remodeling, PAFs become highly activated and undergo phenotypic switching characterized by excessive proliferation, migration, and pro-inflammatory activity.

Adventitial thickening decreases vascular compliance and distensibility, thereby elevating pulmonary pressures and contributing to right-ventricular dysfunction. These adventitial changes are now considered the earliest and most prominent structural abnormalities in PAH [56], preceding intimal and medial remodeling and subsequently contributing to medial degeneration and thickening. Experimental studies demonstrate that hypoxia can double adventitial thickness via hypertrophy and hyperplasia of resident fibroblasts, accompanied by increased collagen deposition. Under hypoxic stimulation, adventitial fibroblasts undergo phenotypic switching, proliferate, and differentiate while upregulating contractile and extracellular-matrix proteins, and release paracrine mediators that influence medial PASMCs. Local hypoxia also increases carbonic anhydrase activity within the adventitia, thereby promoting arterial remodeling and inflammatory responses [57].

Hypoxia induces PAF proliferation and their transition into myofibroblasts. These myofibroblasts migrate from the adventitia toward the intima and media, thickening the vessel wall and serving as major sources of collagen, fibronectin, tenascin, and elastin. Thus, they are recognized as key contributors to pulmonary vascular remodeling [58].

The adventitia plays a pivotal role in the development and progression of PAH. Elucidating its molecular mechanisms in hypoxia-driven vascular remodeling and identifying therapeutic targets within this layer may yield novel strategies for treating PAH.

### 2.2. Endothelial-to-Mesenchymal Transition (EndMT) in PAH

#### 2.2.1. Overview of EndMT in PAH

**Figure 4:**
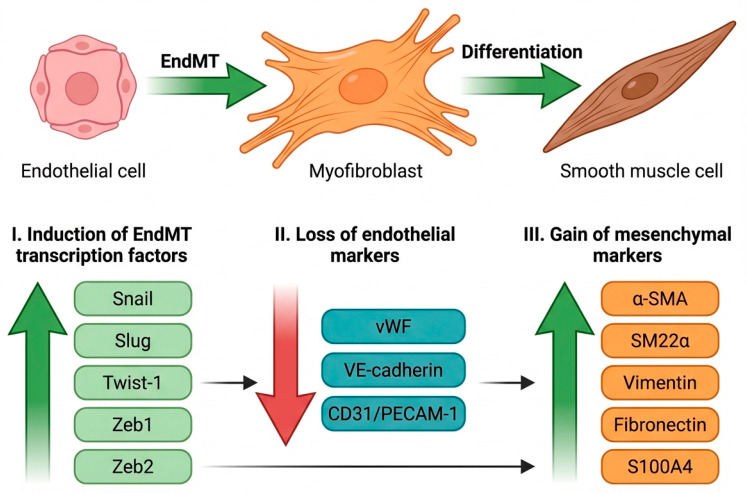
Sequence of events in the course of EndMT progression. Induction of EndMT-associated transcription factors Snai1, Snai2, Twist-1, Zeb1, and Zeb2 leads to progressive loss of endothelial markers PECAM-1 and VE-cadherin, accompanied by gain of mesenchymal markers vimentin, fibronectin, SM22α, and α-SMA. The figure also depicts the sequential phenotypic transition from endothelial cells to myofibroblasts and ultimately to smooth muscle-like cells during EndMT progression. Green arrows denote activation, upregulation, or forward progression of EndMT, including mesenchymal differentiation and marker acquisition, whereas the red downward arrow denotes reduction or loss of endothelial characteristics and marker expression. α-SMA, alpha-smooth muscle actin; EndMT, endothelial-to-mesenchymal transition; PECAM-1, platelet endothelial cell adhesion molecule; SM22α, transgelin; Snai1, Snail; Snai2, Slug; VE-cadherin, vascular endothelial cadherin.

EndMT is a dynamic cellular process regulated by multiple converging signaling pathways. Broadly defined, EndMT refers to the conversion of endothelial cells (ECs) into cells exhibiting a mesenchymal-like phenotype. Given that this transition involves numerous intermediate states and variable endpoints, clearly delineating a precise temporal sequence of signaling events has proven challenging [14]. Analogous to epithelial-to-mesenchymal transition (EMT), the initiation of EndMT involves activation of multiple transcription factors, notably the zinc-finger proteins Snail (SNAI1) [59], Slug (SNAI2) [60], Zeb1 [61], Zeb2 [62], and Twist-1 [63]. Initially characterized in EMT [64,65,66], these factors function as repressors or activators of endothelial and mesenchymal gene expression programs. They orchestrate the loss of endothelial-specific proteins, including von Willebrand factor (vWF), platelet-endothelial cell adhesion molecule-1 (PECAM-1/CD31), vascular endothelial cadherin (VE-cadherin), vascular endothelial growth factor receptor (VEGFR), and angiopoietin receptor (Tie-2), while simultaneously driving expression of mesenchymal markers such as S100 calcium-binding protein A4 (S100A4/FSP-1), α-smooth muscle actin (α-SMA), transgelin (SM22α), fibronectin (FBN), and vimentin [67]. Ultimately, transcriptional reprogramming during EndMT, driven by cytoskeletal reorganization and ECM remodeling, results in a morphological transition from a characteristic dense, cobblestone-like endothelial cell appearance to an elongated, spindle-shaped mesenchymal phenotype [68,69] (Figure 4).

Since the identification of EndMT as a critical mechanism underlying pulmonary vascular remodeling in PAH, Qiao et al. employed a monocrotaline (MCT)-induced PAH model to conduct cell fate-mapping studies [70]. They examined endothelial-derived cells to establish the cellular origin of pathological neointimal lesions. Immunostaining demonstrated co-localization of α-SMA with GFP-labeled endothelial lineage cells, providing evidence that endothelial cells of pulmonary vessels can undergo EndMT, thereby contributing to neointimal formation under pathophysiological conditions associated with PAH. Moreover, Qiao et al. first reported co-localization of endothelial markers PECAM-1 and vWF with α-SMA within neointimal cells from human PAH patients, highlighting the translational relevance of EndMT in clinical disease. Using a BMPR2-deficient rat strain characterized by pulmonary arterial medial hypertrophy, another study provided the initial link between impaired BMPR2 signaling and EndMT, as evidenced by up-regulation of the transcription factor Twist-1 and mesenchymal marker phospho-vimentin in whole-lung lysates from BMPR2-mutant rats [71].

ET-1, a 21-amino-acid peptide hormone belonging to the endothelin family (ET-1, ET-2, ET-3), is one of the most potent endogenous vasoconstrictors identified. Primarily synthesized and released by vascular endothelial cells, ET-1 secretion remains low under physiological conditions but markedly increases in response to pathological stimuli such as hypoxia, inflammation, or mechanical injury. Pathological release of ET-1 significantly elevates peripheral vascular resistance, contributing to acute hypertensive events, including blood-pressure volatility observed in hypertension and acute myocardial infarction. Through binding to ETA receptors, ET-1 promotes proliferation and migration of vascular smooth muscle cells and cardiac myocytes, and enhances ECM synthesis, leading to vascular wall thickening and lumen narrowing. In a 2025 study, Montezano and colleagues employed a hypoxia-induced PAH mouse model to demonstrate that PAH mice exhibited pulmonary vascular remodeling, endothelial dysfunction, and enhanced ET-1-mediated vasoconstriction, thereby linking elevated pulmonary ET-1 expression and eNOS activation with EndMTT [70]. Despite inherent limitations in animal models and in vitro experiments, accumulating evidence suggests that EndMT and other well-characterized mechanisms of endothelial dysfunction significantly contribute to PAH initiation and progression. Because endothelial injury serves as a critical initiating event in PAH, investigation of EndMT is essential for elucidating disease pathophysiology and holds considerable promise for future therapeutic and preventive strategies. Decades of research have established a robust link between EndMT and PAH in both animal models and human patients. To clarify these relationships further, it is crucial to recognize that systemic and organ-level pathophysiological responses, including hypoxia-induced vascular remodeling, endothelial dysfunction, inflammatory responses, abnormal ECM deposition, and endothelial apoptosis, are integral components of PAH and may drive disease progression. Collectively, these factors result in structural and functional alterations of pulmonary vessels, thus promoting PAH development. Therefore, a detailed investigation of EndMT’s role in these processes is vital for a comprehensive understanding of PAH pathogenesis and may provide critical insights into novel therapeutic strategies.

#### 2.2.2. Drivers and Mechanisms of EndMT in PAH

##### Role of Hypoxia in PAH-Associated EndMT

Although chronic hypoxia alone can induce PAH even in the absence of other pathological insults, hypoxia in the lung typically coexists with severe vascular occlusion and remodeling characteristic of various forms of PAH, particularly group III PH [72]. Despite evidence linking hypoxia to EndMT in pulmonary arterial, microvascular, and macrovascular endothelial cells, mechanistic studies directly connecting hypoxia to EndMT in PAH remain limited.

The effects of hypoxia on peripheral tissues are primarily mediated by hypoxia-inducible factors (HIFs), specifically HIF-1α and HIF-2α. Among the three known HIF-α isoforms, HIF-1α, HIF-2α, and HIF-3α, HIF-1α is ubiquitously expressed, while HIF-2α expression is restricted to specific cell types, including endothelial cells, cardiomyocytes, hepatocytes, type II alveolar epithelial cells, glial cells, and renal cortical interstitial cells. HIF-3α remains less well characterized and is currently considered an endogenous inhibitor of HIF-1α and HIF-2α. Distinct HIF-α subunits regulate different gene sets: HIF-1α mainly controls glycolytic enzyme expression, whereas HIF-2α regulates OCT-4, EPO, NDRG1, VEGF, and other genes [73].

Emerging evidence indicates that both the duration and intensity of hypoxia act as “switches” governing the extent and persistence of HIF-1α and HIF-2α activation. Under variable hypoxic conditions, these two isoforms are stabilized for distinct periods and engage in separate yet complementary physiological and pathological processes. They bind hypoxia-response elements (HREs) within hypoxia-inducible genes, transactivating their expression [73]. Recent studies [74] have established a causal chain linking hypoxic exposure, induction of specific HIF isoforms, and EndMT, highlighting the role of HIF-1α in upregulating the EndMT transcription factor Twist-1. Zhang et al. [74] demonstrated direct binding of HIF-1α to the Twist-1 promoter in rat pulmonary microvascular endothelial cells (PMVECs). They further showed that HIF-1α knockdown partially reversed the hypoxia-induced decrease in CD31 and increase in α-SMA and collagen type 1α1 (Col1a1). Importantly, silencing HIF-1α also reversed hypoxia-induced upregulation of Twist-1, suggesting a mechanistic link between HIF-1α signaling and EndMT.

A study by Tang et al. [75] further clarified the mechanistic relationship between hypoxia and EndMT in PAH. They observed elevated expression of HIF-2α in pulmonary vascular endothelial cells (PVECs, including PAECs and PMVECs) isolated from idiopathic PAH (iPAH) patients. Compared to cells from non-PAH lungs, iPAH PVECs showed increased mRNA levels of EndMT transcription factors Snail and Slug and mesenchymal markers α-SMA, S100A4, and fibronectin, accompanied by reduced endothelial markers PECAM-1 and VE-cadherin, indicating active EndMT in iPAH pulmonary vasculature. Interestingly, silencing HIF-2α rather than HIF-1α in iPAH PVECs reduced the expression of Snail and Slug, highlighting HIF-2α as the critical driver of EndMT under hypoxic conditions. Furthermore, endothelial-specific deletion of HIF-2α, but not HIF-1α, prevented hypoxia-induced PAH in mice. It remains unclear whether the loss of HIF-2α can reverse hypoxia-driven EndMT and how endothelial HIF-1α contributes to PAH-associated EndMT. The apparent discrepancy, HIF-1α regulating Twist-1 versus HIF-2α regulating Snail and Slug, may arise from technical variations among laboratories or species-specific responses, necessitating further investigation. Several studies have indicated that hypoxia may also regulate EndMT progression via microRNAs (miRNAs) [74]. Liu et al. recently demonstrated that miR-27a contributes to hypoxia-induced, PAH-related EndMT by targeting the transcriptional repressor DNA-binding inhibitor 2 (Id2) [74]. In addition to elevated expression in pulmonary arteries of chronically hypoxic rats, miR-27a was upregulated in human PAECs exposed to hypoxia in vitro. Functional studies showed that miR-27a inhibition reversed hypoxia-induced EndMT, whereas miR-27a overexpression promoted EndMT under normoxic conditions, as evidenced by reciprocal changes in CD31, α-SMA, and vimentin expression. Furthermore, this group demonstrated that miR-27a suppresses Smad5, a critical mediator of BMP signaling. They proposed that miR-27a-mediated downregulation of Smad5 reduces Id2 expression, an endogenous inhibitor of Snail and Twist-1, thus promoting EndMT.

Finally, recent evidence highlighting hypoxia-mediated regulation of EndMT has identified an additional layer involving galectins, a family of soluble β-galactoside-binding lectins [76]. These proteins have increasingly been implicated in chronic and acute inflammatory responses underlying cardiovascular diseases [77] and are clinically recognized as serological prognostic biomarkers in chronic heart failure patients [78]. Studies indicate that galectin-3 mediates PDGF signaling in PH [79] and actively promotes pulmonary vascular remodeling [80]. In addition, genetic deletion of galectin-3 protects mice from hypoxia-induced PAH [81]. Zhang et al. further clarified galectin-3’s role in PAH and EndMT, demonstrating that hypoxia-induced galectin-3 upregulation promotes α-SMA expression in pulmonary endothelial cells via the Jagged-1/Notch-1 signaling pathway [76].

Collectively, these findings illustrate the mechanisms by which hypoxia drives EndMT in PAH, highlighting critical roles for HIF signaling and potential involvement of miRNAs and galectins. Twist-1, Snail, and Slug appear to be important downstream targets mediating EndMT, and future research will likely further delineate precise molecular pathways underlying hypoxia-induced EndMT in PAH-related endothelial pathology. In addition to galectin-3, emerging evidence suggests that galectin-8 also participates in angiogenesis [82], cell adhesion, and proliferation [83]; therefore, its potential role in PAH pathogenesis and EndMT warrants further investigation. Although research linking miRNAs to EndMT in PAH remains limited, available studies underscore the relevance of these regulatory molecules and support further exploration.

##### Impact of Immune Dysregulation and Inflammatory Responses on PAH-Related EndMT

Histopathological examinations of lungs from PAH patients strongly support inflammation as a critical pathogenic driver of the disease. Several lines of evidence indicate that inflammatory responses precede vascular remodeling, suggesting that immune dysregulation is a cause, rather than a consequence, of vascular pathology [84].

Inflammation represents a fundamental element in the pathogenesis of all forms of PAH, including human PAH and hypoxia-induced PAH animal models [85,86,87], and is regarded as a primary driver of pulmonary vascular remodeling. PAH-associated inflammation is characterized by infiltration of innate and adaptive immune cells into pulmonary arterial walls, especially at sites of vascular lesions, accompanied by increased levels of circulating and local cytokines and chemokines [88]. Perivascular inflammatory infiltrates in PAH typically include macrophages, mast cells, T and B lymphocytes, and dendritic cells [89]. Upon activation, these cells release inflammatory mediators that upregulate signaling proteins and amplify inflammatory cascades, contributing significantly to PAH progression [90]. Previous studies demonstrated that cytokines such as interleukin-1β (IL-1β), interleukin-6 (IL-6), leukotriene B4 (LTB4), and tumor necrosis factor-α (TNF-α) play pivotal roles in pulmonary vascular remodeling. For instance, IL-6 overexpression activates the signal transducer and activator of transcription 3 (STAT3) pathway and Krüppel-like factor 5, promoting vascular remodeling [91]. LTB4 attenuates endothelial sphingosine-1-phosphate (S1P) signaling by reducing sphingosine kinase 1 expression, thereby decreasing S1P levels and impairing eNOS activation, which ultimately leads to endothelial dysfunction and pulmonary vascular remodeling [92]. TNF-α contributes to mitochondrial membrane hyperpolarization and activates nuclear factor of activated T cells (NFAT) signaling by inhibiting pyruvate dehydrogenase activity, shifting smooth-muscle cells toward apoptosis-resistant phenotypes that foster pulmonary vascular remodeling [93]. Patients with PAH display impaired regulatory T-cell function and systemic and pulmonary immune activation. In addition to disturbed T and B cell activities, these patients exhibit enhanced neutrophil elastase activity and macrophage activation [94]. Macrophages play critical roles in numerous pulmonary diseases; notably, pulmonary fibrosis is closely associated with M2 macrophages that produce pro-fibrotic chemokines (e.g., CCL-17, CCL-18, and CCL-23) and various matrix remodeling enzymes [95]. Autopsies of fatal cases involving severe acute respiratory syndrome coronavirus (SARS-CoV-1), Middle East respiratory syndrome coronavirus (MERS-CoV), and SARS-CoV-2 consistently demonstrated extensive infiltration by macrophage-dominated inflammatory cells in lung tissues [96]. In patients experiencing severe SARS-CoV-2-induced respiratory failure, macrophage hyperactivation and immune dysregulation have been well documented. Macrophages represent approximately 70% of total pulmonary leukocytes [97] and can sustain infection by numerous viruses, including respiratory syncytial virus, enabling viral replication and prolonged persistence within pulmonary tissues [98].

##### Impact of Oxidative Stress and Redox Imbalance on PAH-Related EndMT

In addition to hypoxic and pro-inflammatory stimuli, pulmonary vessels in PAH exhibit increased production of ROS and reactive nitrogen species (RNS), resulting in heightened susceptibility to oxidative injury during vascular remodeling.

Under physiological and pathological conditions, major enzymatic sources of ROS and RNS include iron-derived redox systems (heme and iron–sulfur clusters), NADPH oxidases (Nox isoforms), mitochondrial electron-transport complexes I–III, uncoupled nitric oxide synthases (NOS), and xanthine oxidase (XO). Antioxidant defenses comprise catalase, superoxide dismutases (SODs), the thioredoxin system, glutathione peroxidases, peroxiredoxins, and antioxidant vitamins C and E. The primary ROS, superoxide (O_2_^−^) and its dismutation product hydrogen peroxide (H_2_O_2_), are elevated in nearly all pulmonary vascular cell types exposed to PAH-relevant stimuli and exert direct pathogenic effects. Likewise, RNS such as peroxynitrite (ONOO^−^), generated from nitric oxide oxidation, contribute to PAH pathophysiology. In affected patients, levels of nitric oxide and its metabolites (NO_2_^−^, NO_3_^−^, S-nitrosothiols) are reduced in lung tissue. Increasing these vasodilatory species has been postulated to exert protective effects. ROS promote PAH development by impairing nitric-oxide-mediated vasodilation and inducing a proliferative PASMC phenotype through ERK signaling activation [99].

Multiple groups have reported elevated superoxide production by endothelial Nox2 (gp91phox) in PAH. Protective effects observed in Nox2-deficient mice, including mitigation of PH-related pathological changes and improved NO-mediated vasorelaxation, indicate that Nox2-derived ROS play a pivotal role in hypoxia-induced PAH [100,101]. Additional studies show increased Nox4 expression in adventitial, endothelial, and smooth muscle cells under hypoxia in vitro and in PAH animal models [102,103,104]. Accumulating evidence for the pathogenic role of ROS in PAH has motivated the development of antioxidant-based therapeutic strategies. Superoxide dismutase mimetics [105], extracellular SOD gene delivery [106], the glutathione precursor N-acetylcysteine [107], the xanthine-oxidase inhibitor allopurinol [108], and pharmacological Nox4 inhibition [102] have all shown protective effects in hypoxia-induced and monocrotaline PAH models. However, despite strong preclinical efficacy, clinical outcomes have been disappointing; for example, dietary supplementation with the mitochondria-targeted antioxidant coenzyme Q10 failed to improve hemodynamics in PAH patients. Intriguingly, some animal studies suggest that ROS may also support adaptive processes in PAH [109], which could partly explain the discrepancy between preclinical benefits and limited clinical efficacy.

Although oxidative stress remains a hallmark of PAH vasculature [110], and ROS upregulation is causally linked to PAH development, the relationship between oxidative stress and PAH-related EndMT is only beginning to be explored.

##### Impact of Endothelial Metabolism on PAH-Related EndMT

EndMT is normally initiated by TGF-β family ligands and is essential during cardiac valve development. When dysregulated, it contributes to pathological processes including atherosclerosis, PAH, and fibrosis. Xiong et al. [111] demonstrated both in vivo and in vitro that genetic deletion or pharmacologic inhibition of carnitine palmitoyltransferase 2 (CPT2), which reduces endothelial fatty acid oxidation (FAO), exacerbates EndMT severity. CPT2 is a rate-limiting enzyme in FAO, responsible for transporting long-chain fatty acyl-CoA from the cytosol into mitochondria for β-oxidation. CPT2 knockdown or deletion lowers FAO, leading to reduced intracellular acetyl-CoA, a key metabolic intermediate. Reduced acetyl-CoA disturbs multiple processes, including cholesterol biosynthesis and protein acetylation. Simultaneously, decreased acetyl-CoA impairs SMAD7 signaling, a critical negative-feedback regulator that restrains TGF-β-driven EndMT. Thus, CPT2 loss, via reduced FAO, decreased acetyl-CoA, and impaired SMAD7 activity, amplifies EndMT, identifying a novel therapeutic entry point in diseases where EndMT is pathogenic. Collectively, these findings highlight the central importance of metabolic regulation in controlling EndMT.

## 3. Therapeutic Outlook for Targeting EndMT in PAH

Given the refractory nature and poor prognosis of PAH, novel therapeutic approaches are urgently required to improve patient outcomes and quality of life. Emerging evidence underscores the pivotal role of EndMT in pulmonary vascular remodeling and PAH progression, suggesting that strategies designed to reverse EndMT represent promising therapeutic avenues. Although research specifically targeting EndMT in PAH remains limited, recent preclinical studies indicate that mesenchymal stem cell (MSC) therapy [112], dipeptidyl peptidase-4 (DPP-4) inhibition [113], and CD44 blockade effectively ameliorate PAH by modulating EndMT pathways. MSC transplantation is known to reduce RV pressure, hypertrophy, and inflammation in animal models of PAH. Furthermore, EndMT has been recognized as an essential aspect of endothelial dysfunction both in human PAH and animal models, including monocrotaline [114] and chronic hypoxia [11]. Nevertheless, only a few studies have specifically investigated MSC-based therapies targeting EndMT in PAH. Huang et al. [112] demonstrated that intravenous MSC administration significantly decreased RV systolic pressure, RV hypertrophy, and pulmonary vascular muscularization in hypoxic rats. MSC treatment also attenuated collagen deposition and normalized the expression of ECM metalloproteinases (MMP2 and MMP9). Importantly, MSC administration reduced the hypoxia-induced co-localization of α-SMA and vWF in pulmonary vessels, suggesting potential reversal of EndMT. These in vivo findings are supported by in vitro observations where exposure of pulmonary microvascular endothelial cells to MSC-conditioned medium reversed hypoxia-induced HIF-2α accumulation [75]. Nevertheless, these studies primarily demonstrated correlation rather than causation, and the absence of appropriate MSC-free control groups limits their mechanistic conclusions.

DPP-4 (CD26), a serine protease modulating chemokines, cytokines, and vasoactive peptides, has emerged as a promising therapeutic target for suppressing EndMT in PAH [113]. DPP-4 is abundantly expressed on the plasma membranes of various cell types, including pulmonary capillary endothelial cells [115]. Clinically, DPP-4 inhibitors are employed to treat type 2 diabetes due to their glucose-lowering effect by preventing glucagon-like peptide-1 (GLP-1) degradation [116]. Beyond their antihyperglycemic properties, recent studies highlight their cardiovascular protective actions and potential to reverse pulmonary EndMT in models of endotoxin-induced acute lung injury [117]. Xu et al. demonstrated that administration of the DPP-4 inhibitor sitagliptin in monocrotaline-induced PAH rats reduced serum DPP-4 levels and significantly decreased α-SMA/CD31 double-positive cells within pulmonary arterial walls. Additionally, sitagliptin treatment suppressed lung expression of mesenchymal markers α-SMA, vimentin, and fibronectin, while restoring endothelial markers VE-cadherin and vWF [117].

A recent study explored pathophysiological similarities between PAH and cancer, linking the adhesion molecule variant CD44v8-10 to EndMT and PAH [118]. Remodeled pulmonary vascular endothelial cells (ECs), like cancer cells, display increased proliferation and apoptosis resistance. Notably, Isobe et al. detected CD44v expression in EndMT-derived cells within neointimal lesions from PAH patients and in chronic hypoxia-exposed mouse lungs [119], supporting the involvement of CD44 in EndMT. Stimulation of human pulmonary artery endothelial cells (HPAECs) with TGF-β, TNF-α, and IL-1β to induce EndMT markedly elevated CD44 and CD44v8-10 expression, accompanied by increased levels of the transcription factor Snail, reduced CD31 expression, and upregulation of mesenchymal markers α-SMA, SM22α, MMP2, and MMP9. In addition, a subset of CD44v8-10-positive HPAECs highly expressed the cystine–glutamate antiporter subunit xCT, enhancing intracellular glutathione (GSH) synthesis and conferring oxidative-stress resistance similar to cancer cells. Collectively, these findings suggest CD44v involvement in PAH-related EndMT, highlighting the therapeutic potential of targeting CD44 and xCT in PAH. Current PAH therapies might also indirectly impact EndMT. Given the established connection between ET-1 signaling and EndMT in PAECs [114], endothelin receptor antagonists such as bosentan could potentially confer additional therapeutic benefits through EndMT suppression [120]. Moreover, substantial evidence linking dysregulated BMPR2 signaling and EndMT in PAH suggests that developing BMPR2-targeted therapies may also mitigate pulmonary vascular EndMT. However, antioxidant therapies require cautious evaluation due to their potential to inadvertently promote EndMT.

Beyond the aforementioned approaches involving DPP-4 inhibitors, CD44 blockade, and MSC-based treatments, other mediators of EndMT detailed herein, such as specific microRNAs, galectin-3, and HMGA1, could be targeted to restore BMPR signaling and redox homeostasis in the pulmonary vasculature, representing promising therapeutic strategies for reversing EndMT and reducing PAH-related vascular remodeling. Indeed, accumulating evidence on EndMT’s role and increasing mechanistic insights position this process as an attractive therapeutic target in future PAH interventions.

## 4. Conclusions

A comprehensive understanding of vascular remodeling and the mechanisms driving PAH is critical for developing effective anti-remodeling therapies. Mounting evidence indicates that EndMT significantly contributes to PAH pathogenesis through its integration of diverse environmental and signaling inputs. Hypoxia, BMPR2 dysregulation, inflammation, and oxidative stress have all emerged as pivotal triggers of EndMT in PAH. Despite this evidence, the mechanisms underlying EndMT in PAH remain incompletely characterized, necessitating further investigation to clarify its role in disease progression. Identifying novel therapeutic targets is particularly important, given the limitations and adverse side effects of existing therapies.

It should be emphasized that although the studies reviewed have established a foundational understanding of EndMT in PAH, current experimental methods carry significant limitations. For instance, many studies evaluating EndMT markers or transcription factors have relied on whole-lung homogenates rather than isolated intimal or neointimal cell populations, potentially reflecting broader fibrotic responses or EMT. Furthermore, markers commonly employed for endothelial lineage tracing, such as CD31, can also be expressed by other cell types, raising concerns of specificity. Moreover, the complexity of EndMT and the absence of standardized criteria for endothelial or mesenchymal phenotypes hinder comparative analysis across studies. To overcome these limitations, enhanced data sharing, interdisciplinary collaboration, and increased use of endothelial-specific lineage tracing and genetic models are required. Emerging technologies, including single-cell RNA sequencing and super-resolution microscopy, promise to deepen our understanding of transitional endothelial phenotypes in PAH patients, facilitating the identification of novel therapeutic targets.

Although several candidate therapies, such as dichloroacetate, BMPR2 modulators, ROCK inhibitors, and HIF inhibitors, have shown promising results in preclinical studies, large-scale clinical trials are essential to establish their efficacy in human PAH. Major challenges persist, particularly in translating preclinical findings into tangible clinical benefits, a long-standing obstacle in PAH research. Further studies into the mechanisms and regulatory pathways governing pulmonary vascular remodeling are essential to provide a robust theoretical framework for the development of innovative, targeted therapeutic strategies aimed at reversing remodeling and improving outcomes in PAH.

## Data Availability

All data cited in this review are derived from previously published literature, and the relevant references are listed in the bibliography at the end of the manuscript.
